# Association of mNUTRIC and NRS 2002 Scores with Mortality in Intensive Care Unit Patients

**DOI:** 10.12669/pjms.41.7.12087

**Published:** 2025-07

**Authors:** Yildirım Ar Arzu, Bal Keske Pelin, Alagoz Sevgi, Turan Guldem

**Affiliations:** 1 Yıldırım Ar Arzu, MD Associate Professor, Clinic of Anesthesiology and Reanimation, University of Health Sciences, Fatih Sultan Mehmet, Teaching and Research Hospital, Istanbul, Turkey; 2 Bal Keske Pelin, MD Clinic of Intensive Care, University of Health Sciences, Siyami Ersek, University of Health Sciences, Fatih Sultan Mehmet, Teaching and Research Hospital, Istanbul, Turkey, Thoracic and Cardiovascular Surgery Teaching and Research Hospital, Istanbul, Turkey; 3 Alagoz Sevgi, MD Clinic of Anesthesiology and Reanimation, University of Health Sciences, Fatih Sultan Mehmet, Teaching and Research Hospital, Istanbul, Turkey; 4 Turan Guldem, MD Professor, Clinic of Intensive Care, University of Health Sciences, Basaksehir Cam And Sakura City, Teaching and Research Hospital, Istanbul, Turkey

**Keywords:** Intensive Unit, Mortality, Nutrition Assessment

## Abstract

**Background & Objectives:**

In intensive care unit (ICU) patients, comorbidities, age, and nutritional status may affect mortality and different scores assess nutritional status. The Nutrition Risk in Critically Ill (NUTRIC) score incorporates IL-6, age, APACHE II, SOFA score, the number of comorbidities, and time to ICU admission. A high score (6-10) indicates a high malnutrition risk. The modified NUTRIC Score (mNUTRIC score) does not include IL-6 and scores of 5-9 indicate a high malnutrition risk. In the Nutritional Risk Screening (NRS 2002) score, malnutrition is graded as none, mild, moderate, or severe, and a score of ≥3 indicates malnutrition risk. Our objective was to examine the association of mortality with the mNUTRIC and NRS 2002 scores on the first day of admission to the ICU.

**Methods:**

Our observational, prospective study was conducted with 50 ICU patients between November 2018 to January 2019 mNUTRIC and NRS 2002 scores were recorded at admission and their associations with mortality were analyzed. The duration of mechanical ventilation and length of ICU stay were compared between high and low risk patients.

**Results:**

The NRS 2002 and mNUTRIC scores did not differ in their association with mortality. The high-risk group according to the NRS 2002 had significantly longer(p=0.048) mechanical ventilation duration than the low-risk group.

**Conclusion:**

The mNUTRIC score was used instead of the NUTRIC score due to infeasibility of IL-6 monitoring. The NRS 2002 and mNUTRIC scores did not differ in their association with mortality. Both scores may be recommended for routine use in the ICU.

## INTRODUCTION

Comorbidities, age, and nutritional status may affect the mortality of intensive care unit (ICU) patients. Different scores are used in intensive care settings to evaluate the nutritional status of patients.^1^ The Nutrition Risk in Critically III (NUTRIC) score developed by Heyland et al. was the first nutritional risk score developed for intensive care patients.^2^ Although there are many scores for nutritional screening and risk assessment for intensive care patients, the NUTRIC score is highly specific for such patients. To predict the 28-day mortality, the NUTRIC score incorporates acute fasting, chronic fasting, acute and chronic inflammatory markers (including interleukin (IL)-6), age, Acute Physiology and Chronic Health Evaluation (APACHE) II score, Sequential Organ Failure Assessment score (SOFA), number of comorbidities, and time from hospital admission to ICU admission. A high score (6-10) indicates a high mortality risk. The parameters used in the NUTRIC score, except for IL-6, are used in our daily practice. Because routine monitoring of IL-6 is not always possible, the modified NUTRIC Score (mNUTRIC score) was introduced in 2016 as a variation of the NUTRIC score that does not include IL-6, with a score of 5-9 indicating high nutritional risk and scores between 0-4 indicating low risk.^3^,^4^

Nutritional Risk Screening 2002 (NRS-2002), first described by Kondrup et al., is a screening tool used to detect the presence of malnutrition and predict the risk of malnutrition in hospitalized patients.^5^ The NRS-2002 score consists of two stages. In the first stage, body mass index (BMI, < 20.5), food intake in the previous week, weight loss in the last three months, and the presence of severe disease were assessed. The assessment was continued if one of these four questions was answered positively. Disease severity and deterioration in nutritional status were assessed using a score between zero and three, based on whether they were absent, mild, moderate, or severe. The total score was calculated by adding the score gained from age to the rest of the scores. The total score ranged from zero to seven, with a score ≥ 3 defining nutritional risk.^6^

In this prospective study, we investigated the relationship between mortality and mNUTRIC and Nutritional Risk Score (NRS 2002) scores on the first day of admission. The mNUTRIC score, rather than the NUTRIC score, was used because of the time and economic cost of IL-6 measurement. Since other factors affect mortality in ICU patients, the APACHE II score was used to standardize the patients.

## METHOD

This prospective observational study was conducted at the University of Healty Sciense Fatih Sultan Mehmet Health Application and Research Center Anesthesiology and Reanimation Clinic. With consent from the patients’ relatives, 50 patients with an ICU stay of at least 24 hour between November 10, 2018 to January 10, 2019 were included in the study. Our clinic is a 20-bed tertiary ICU. Indications for hospitalization were classified as primary respiratory failure, secondary respiratory failure, neurological causes, postoperative patients, cardiopulmonary resuscitation, or traumatic causes. Demographic data, mean APACHE II score, Simplified Acute Physiology Score (SAPS 2), mNUTRIC Score, NRS 2002, length of hospitalization, duration of invasive or non-invasive mechanical ventilation (MV), and mortality were recorded. The mNUTRIC and NRS 2002 scores were recorded by intensive care doctor on the day of hospitalization and their associations with mortality were analyzed. Scores were calculated based on admission data and clinical status. The durations of ICU stay and MV were also compared between low- and high-risk patients for both scores.

### Ethical Approval:

The study was approved by the hospital Ethics Committee (FSMEAH-KAEK 2018/64; Dated: October 25, 2018).

### Statistical Analysis:

SPSS Statistics (version 22.0; IBM, Armonk, NY, USA) was used for statistical analysis. The normal distribution of parameters was tested using the Shapiro-Wilk test, and no parameter had a normal distribution. In addition to descriptive statistics (mean, standard deviation, median, and frequency), the Mann-Whitney U test was used to compare the quantitative data between the two groups. Fisher’s exact chi-square test with Yates’ continuity correction was used to compare qualitative data. The discriminatory ability of the scores for mortality was assessed using receiver operating characteristic curve (ROC) analysis. Statistical significance was set at p<0.05.

## RESULTS

This study included 50 patients, 27 (54%) of whom were female and 23 (46%) were male. The mean age of the study patients was 67.74 ± 17.44 years. The mean duration of hospital stay was 13.94 ± 12.43 days and the mean duration of MV was 8.94 ± 12.25 days. The mean NRS 2002 score was 3.6 ± 1.2 and the mean mNUTRIC score was 4.34 ± 1.98. The mean APACHE II, SAPS II, and SOFA scores are shown in Table-I. The indications for ICU admission were secondary respiratory failure (36% of patients), neurological causes (24%), postoperative (16% of cases), and primary respiratory failure (12% of the patients). Mortality was observed in 12 cases (24%). According to the NRS 2002 score, 84% of the patients were at a high risk of malnutrition, while 48% were at high risk according to the mNUTRIC score (Table-I).

**Table-I:** Age, length of stay, duration of mechanical ventilation, risk scores, indications for admission, and mode of discharge of study patients.

	Min-Max	Mean ± SD	Median
Age	23-98	67.74±17.44	71
Length of stay (days)	1-56	13.94±12.43	12
Duration of mechanical ventilation (days)	0-56	8.94±12.25	5
NRS 2002	1-6	3.6±1.2	4
mNUTRİC	1-9	4.34±1.98	4
APACHE II	5-39	17.28±7.71	17
SAPS II	13-72	37.36±15.25	35.5
SOFA	0-15	4.84±3.1	4

		***n***	***%***

Indication for admission	Primary respiratory failure	6	12
	Secondary respiratory failure	18	36
	Neurological diseases	12	24
	Postoperative causes	8	16
	Cardiopulmonary resuscitation	2	4
	Trauma	4	8
NRS 2002	Low risk	8	16
	High risk	42	84
mNUTRIC	Low risk	26	52
	High risk	24	48
Mode of discharge	Transfer to ward	38	76
	Mortality	12	24

NRS 2002: Nutritional Risk Score 2002, mNUTRİC: modified NUTRIC Score, APACHE II: Acute Physiology and Chronic Health Evaluation II, SAPS II: Simplified Acute Physiology Score II, SOFA: Sequential Organ Failure Assessment score.

Patients at a high risk of malnutrition according to the NRS 2002 score had a mortality rate of 26.2%, while the low-risk patients had a rate of 12.5%, with no statistically significant difference (p>0.05). Similarly, according to the mNUTRIC score, the mortality rate was 33.3% among high-risk patients and 15.4% among low-risk patients, with no significant difference (p>0.05) (Table-II).

**Table-II:** Evaluation of NRS 2002 and mNUTRIC scores for mortality, length of intensive care unit stay and duration of mechanical ventilation.

		Mortality		p

		(+)	(-)	

		n	%	
NRS 2002	Düşük risk	7 (%87.5)	1 (%12.5)	^1^0,661
	High risk	31 (%73.8)	11 (%26.2)	
mNUTRIC	Low risk	22 (%84.6)	4 (%15.4)	^2^0,249
	High risk	16 (%66.7)	8 (%33.3)	

		*ICU stay*	*MV Duration*	

		*Mean ± SD (median)*	*Mean ± SD (median)*	
NRS 2002	Low risk	7.5±6.88 (4.5)	3.5±5.18 (1.5)	
	High risk	15.17±12.91 (12)	9.98±12.96 (5.5)	
	p	^3^0.067	^3^0.048*	
mNUTRIC	Low risk	11.04±10.66 (8.5)	6.35±10.04 (3)	
	High risk	17.08±13.62 (12.5)	11.75±13.94 (8)	
	p	^3^0.063	^3^0.083	

ICU: Intensive Care Unit, MV: Mechanical Ventilation,

1 Fisher’s Exact Test,

2 Yates Continuity Correction,

3 Mann Whitney U Test

* p<0.05 NRS 2002: Nutritional Risk Score 2002, mNUTRIC: modified NUTRIC Score.

The duration of MV was significantly longer in the high-risk group than that in the low-risk group according to the NRS 2002 score (p=0.048; p<0.05), although the duration of ICU stay was longer in the high-risk group than that in the low-risk group, without statistical significance. Both the duration of MV and ICU stay were longer in patients who were at high risk according to the mNUTRIC score than those in the low-risk group; however, this difference was only near-significant (p>0.05) (Table-II).

When the discriminatory ability of the NRS 2002 and mNUTRIC scores for mortality was assessed using ROC analysis, the area under the curve was 0.692 for mNUTRIC and 0.576 for NRS 2002, without a statistically significant difference between the two scores (p>0.05) (Table-III).

**Table-III:** ROC analysis for the discriminatory ability of NRS 2002 and mNUTRIC scores for mortality.

	AUC	SE	95% CI	p
mNUTRIC	0.692	0.0814	0.545 to 0.815	0.185
NRS 2002	0.576	0.0914	0.428 to 0.714	

*AUC: Area Under Curve* NRS 2002: Nutritional Risk Score 2002, mNUTRIC: modified NUTRIC Score.

## DISCUSSION

In our study, we examined the relationship between mortality and the two nutritional risk scores (NRS) on the first day of ICU admission and found no significant difference in the discriminatory abilities between the mNUTRIC and NRS 2002 scores. The duration of MV was significantly longer in patients with high NRS 2002 scores than that in patients with low scores. In their retrospective study of 384 intensive care patients, Machado dos Reis et al. examined the predictive power of the mNUTRIC and NRS 2002 scores alone and in combination for hospital mortality and found that mNUTRIC and NRS-2002, either alone or in combination, performed equally well in predicting mortality and that the mNUTRIC score had a better discriminatory ability to predict the mortality risk in critically ill patients.^7^ Saseedharan et al. evaluated the NUTRIC score, NRS 2002, and Subjective Global Assessment (SGA) in 348 ICU patients and stated that mortality was best predicted by the NUTRIC score, but this study was inconclusive with regard to the screening tools that should be used routinely in the ICU to identify patients at risk of malnutrition. The authors have also stated that their study provides an impetus to design a new screening tool that would be in close agreement with all three commonly used screening tools examined in this study.^8^

In another study conducted by Gülsoy et al. on 311 patients treated in the ICU for more than seven days and who received MV for more than 48 hours, they found a high nutritional risk of 20.9% according to the NRS-2002 score and 62.7% according to the mNUTRIC score. They showed that the risk of in-hospital mortality was three times higher in patients classified as having high nutritional risk according to the mNUTRIC score and two times higher in patients with high nutritional risk according to the NRS-2002 score. They found a strong association between a high mNUTRIC score and inadequate calorie intake, but no association between the mNUTRIC score and protein intake. They stated that the mNUTRIC score was useful for predicting 28-day in-hospital survival, whereas the NRS-2002 did not predict mortality in the same study.^9^

A study by İleri et al., which examined the accuracy of mNUTRIC and NRS-2002 scores in predicting hospital and long-term mortality and the effects of malnutrition on ICU mortality in 81 patients with hematologic malignancy, showed that a high risk of malnutrition was associated with higher ICU mortality rates according to the NRS-2002 score. They found that the NRS-2002 score was superior to the mNUTRIC score in predicting ICU mortality in patients with hematological malignancies. Neither the mNUTRIC nor the NRS-2002 scores were superior to each other in predicting long-term mortality. İleri et al. stated that their results should not be generalized as their study was single-centered and performed only in patients with hematologic cancer.^10^

Coruja et al. in a retrospective cohort study of male-predominant patients (n=208) aged ≥18 years, hospitalized in the ICU for more than 24 hours in two centers in Southern Brazil, aimed to compare nutritional risk as determined by the NUTRIC and the NRS 2002 scores and to determine whether both scores are equivalent for clinical practice in the ICU. They concluded that despite the ability of both scores to identify patients at high nutritional risk, the NUTRIC and NRS 2002 scores had different performances and their risk categorizations were not compatible; therefore, the two risk scores were not equivalent for clinical practice in the ICU.^11^

Our study prospectively examined the relationship between mortality and the mNUTRIC and NRS 2002 scores in patients with ≥ 24 h ICU stay and found no difference between the two scores. Both scores are recommended for evaluating the nutritional risk in intensive care settings. Other studies reached different conclusions from those of the present study. We believe that different results can be achieved with a larger number of patients.

Majari et al. conducted a validation study using three different nutritional screening tools (mNUTRIC, NRS 2002, and Malnutrition Universal Screening Tool (MUST) scores) in 440 patients in four different ICUs and two teaching hospitals in Ireland and examined the relationship between nutritional risk scores and prolonged hospitalisation, prolonged MV, and 28-day mortality. They found that critically ill patients with higher m-NUTRIC scores had longer ICU stays, prolonged MV, and increased 28-day mortality. Both m-NUTRIC and NRS-2002 scores were significantly associated with all three outcomes. They concluded that for the Iranian ICU population, the m-NUTRIC score may be a valid tool to identify patients who would benefit from more aggressive nutritional therapy.^12^

In a prospective cohort study, Sheean et al. examined the association of malnutrition prevalence and adverse outcomes with different nutrition scores in patients treated in surgical and medical ICUs in the geriatric patient population and reported a malnutrition prevalence of 23-34%. Compared to the Mini Nutritional Assessment (MNA), NRS 2002 showed the highest sensitivity, whereas the Subjective Global Assessment (SGA) and MNA-short form (MNA-SF) showed higher specificity for malnutrition. They concluded that malnutrition on admission to the ICU was associated with a longer length of hospital stay, a lower propensity to discharge, more frequent need for hospital care, and death.^13^ Mendes et al. in their multicenter observational study of 1143 Portuguese ICU patients associated higher NUTRIC score values with longer length of stay, fewer MV-free days, and higher 28-day mortality.^14^

Moghaddam et al. evaluated the performance of NRS 2002 score, mNUTRIC score, MNA-SF, Controlling Nutritional status (CONUT), and Prognostic Nutritional Index (PNI) in predicting high or low nutritional risk in Iranian ICU patients in a prospective cohort study on 165 patients and also compared them in terms of mortality, organ failure, length of hospital stay, and MV. They stated that most patients in the ICU are at high risk for malnutrition. This study also showed that when compared with other screening tools, NRS 2002 and mNUTRIC score are more effective in predicting clinical outcomes, whereas other screening tools cannot predict the results accurately because they exaggerate the risk of malnutrition.^15^ Küçük et al. evaluated the mortality prediction power of mNUTRIC and NRS 2002 scores, separately and together, in patients admitted to the ICU with acute respiratory failure (ARF). 16

In addition, in their prospective cohort studies, where they examined whether the result changed with the type of respiratory failure (Type-I: hypoxic respiratory failure, type 2: hypercapnic respiratory failure), they accepted a high nutritional risk when there was a score of 5 or more in any of the nutritional screening tools. They used data to estimate 1-month (30-day) and three months (90-day) mortality with multiple logistic regression analysis. mNUTRIC score, presence of inotropic support, Type-I RF and ward admission were identified as independent variables with a significant association with mortality at one and three months. They found the mNUTRIC score to be the variable most strongly associated with mortality in both periods.

In their study, they detected a high nutritional risk in 57.6% of the patients according to the NRS 2002 score, 78.6% according to the mNUTRIC score and 47.6% according to both nutritional screening scores. In their study, they detected a high nutritional risk in 57.6% of the patients according to the NRS 2002 score, 78.6% according to the mNUTRIC score and 47.6% according to both nutritional screening scores. They reported that malnutrition is associated with deterioration of lung mechanical function in both acute and chronic RF. They stated that not including patients with mixed type RF was one of the limitations of their study.^16^ Our results showed that patients with high malnutrition risk according to the NRS 2002 score required longer MV than those with low malnutrition risk. Detection and management of malnutrition is important in patients treated in the ICU for various reasons.

The American Society for Parenteral and Enteral Nutrition (ASPEN) recommends nutritional screening in patients admitted to the ICU and the use of both scores (NRS 2002 and mNUTRIC score)^11^ Multiple factors are effective and important in determining the mortality of patients in the ICU. Although NRS 2002 includes nutritional parameters for the evaluation of nutritional status (BMI <20.5 kg/m2, recent weight loss, decreased food intake, etc.), mNUTRIC score includes acute hunger and inflammation, chronic hunger and inflammation markers such as SOFA, APACHE II, which we routinely use in the ICU, but does not include traditional nutritional parameters. There are studies in the literature examining different nutritional scores for mortality determination in different patient groups. Depending on this, there are different results. We believe that the results of our study, in which we examined the relationship between mortality and two different scores that we routinely use, namely NRS 2002 and mNUTRIC score, in patients admitted to the ICU with different diagnoses, contribute to the literature. There is no single type of patient group, but the existence of different disease groups affects these results.

### Limitation:

Limitations of our study include being a single center, lack of 30-day or longer follow-up for mortality, and small number of patients. Results may vary with different patient numbers. The presence of ICU admissions with different diagnoses and the lack of homogeneous patient groups may also have an impact on mortality.

## CONCLUSION

We examined the relationship between mortality and mNUTRIC and NRS 2002 scores, and found no significant difference between the two scores. For ICU patients, both scores may be used in daily practice in accordance with the preferences of the clinical team.

### Authors Contributions:

**YAA, BKP and GT:** Concept. Study design, literature search

**YAA, BKP, AS and GT:** Design, Analysis. Interpretation, Literature Search and writing.

**YAA, BKP and AS:** Data Collection. Processing. Critical analysis.

All authors have read and approved the final version and are accountable for integrity of the study.
